# Towards Equal Access to Cytoreductive Surgery with Hyperthermic Intraperitoneal Chemotherapy and Survival in Patients with Isolated Colorectal Peritoneal Metastases: A Nationwide Population-Based Study

**DOI:** 10.1245/s10434-024-15131-0

**Published:** 2024-03-07

**Authors:** Roos G. F. M. van der Ven, Teun B. M. van den Heuvel, Koen P. B. Rovers, Simon W. Nienhuijs, Djamila Boerma, Wilhelmina M. U. van Grevenstein, Patrick H. J. Hemmer, Niels F. M. Kok, Eva V. E. Madsen, Philip de Reuver, Jurriaan B. Tuynman, Felice N. van Erning, Ignace H. J. T. de Hingh

**Affiliations:** 1 Department of Research and Development, Netherlands Comprehensive Cancer Organisation (IKNL), Utrecht, The Netherlands; 2 Department of Health Services Research, Faculty of Health, Care and Public Health Research Institute (CAPHRI), Faculty of Health, Medicine and Life Sciences, Maastricht University, Maastricht, The Netherlands; 3 Department of Oncology and Developmental Biology (GROW), Faculty of Health, Medicine and Life Sciences, Maastricht University, Maastricht, The Netherlands; 4 Department of Surgery, Catharina Hospital, Eindhoven, The Netherlands; 5 Department of Surgery, St Antonius Hospital, Nieuwegein, The Netherlands; 6 Department of Surgery, University Medical Center Utrecht, Utrecht, The Netherlands; 7 Department of Surgery, Division of Surgical Oncology, University Medical Center Groningen, University of Groningen, Groningen, The Netherlands; 8 Department of Surgical Oncology, Netherlands Cancer Institute, Amsterdam, The Netherlands; 9 Department of Surgical Oncology, Erasmus MC Cancer Institute, Rotterdam, The Netherlands; 10 Department of Surgery, Radboud University Medical Center, Nijmegen, The Netherlands; 11 Department of Surgery, Amsterdam UMC Location Vrije Universiteit Amsterdam, Amsterdam, The Netherlands

**Keywords:** Treatment variation, HIPEC, Cytoreductive surgery, Referrals, Network

## Abstract

**Background:**

Before 2016, patients with isolated synchronous colorectal peritoneal metastases (PMCRC) diagnosed in expert centers had a higher odds of undergoing cytoreductive surgery with hyperthermic intraperitoneal chemotherapy (CRS-HIPEC) and better overall survival (OS) than those diagnosed in referring centers. Nationwide efforts were initiated to increase awareness and improve referral networks.

**Methods:**

This nationwide study aimed to evaluate whether the between-center differences in odds of undergoing CRS-HIPEC and OS have reduced since these national efforts were initiated. All patients with isolated synchronous PMCRC diagnosed between 2009 and 2021 were identified from the Netherlands Cancer Registry. Associations between hospital of diagnosis and the odds of undergoing CRS-HIPEC, as well as OS, were assessed using multilevel multivariable regression analyses for two periods (2009–2015 and 2016–2021).

**Results:**

In total, 3948 patients were included. The percentage of patients undergoing CRS-HIPEC increased from 17.2% in 2009–2015 (25.4% in expert centers, 16.5% in referring centers), to 23.4% in 2016–2021 (30.2% in expert centers, 22.6% in referring centers). In 2009–2015, compared with diagnosis in a referring center, diagnosis in a HIPEC center showed a higher odds of undergoing CRS-HIPEC (odds ratio [OR] 1.64, 95% confidence interval [CI] 1.02–2.67) and better survival (hazard ratio [HR] 0.80, 95% CI 0.66–0.96). In 2016–2021, there were no differences in the odds of undergoing CRS-HIPEC between patients diagnosed in HIPEC centers versus referring centers (OR 1.27, 95% CI 0.76–2.13) and survival (HR 1.00, 95% CI 0.76–1.32).

**Conclusion:**

Previously observed differences in odds of undergoing CRS-HIPEC were no longer present. Increased awareness and the harmonization of treatment for PMCRC may have contributed to equal access to care and a similar chance of survival at a national level.

Colorectal cancer (CRC) is the third most diagnosed cancer worldwide.^1,2^ More than 5% of all patients with CRC present with peritoneal metastases at the time of diagnosis, and another 5% develop peritoneal metastases during follow-up after curative resection of the primary tumor.^3^ Selected patients with limited peritoneal metastases of colorectal cancer (PMCRC) can be treated by cytoreductive surgery (CRS) combined with hyperthermic intraperitoneal chemotherapy (HIPEC).^4^ During the last 2–3 decades, overall survival (OS) of patients with PMCRC has improved significantly, which may be a result of the increased use of both modern systemic therapy and advanced surgical procedures such as CRS-HIPEC.^5^ However, previous studies showed significant disparities in access to CRS-HIPEC based on hospital of diagnosis.^6,7^

In The Netherlands, all hospitals diagnose and treat patients with colorectal cancer;^8^ however, CRS-HIPEC is regarded as a complex and complication-prone procedure. Therefore, CRS-HIPEC is performed in a restricted number of high-volume Dutch HIPEC centers, similar to practice in several other European countries.^7,9^ As a result, patients are often initially diagnosed with PMCRC in a hospital that does not perform CRS-HIPEC (i.e. referring centers).

A previous Dutch study, based on data up until 2015, revealed that patients with isolated synchronous PMCRC diagnosed in referring centers received CRS-HIPEC 20% less frequently (odds ratio [OR] 3.66, 95% confidence interval [CI] 2.40–5.58) and experienced worse OS than patients diagnosed in HIPEC centers (9.6 months vs. 14.1 months; hazard ratio [HR] 0.82, 95% CI 0.67–0.99).^7^ The results of this study initiated nationwide efforts, encompassing education, the inclusion of CRS-HIPEC in guidelines, the establishment of a national multidisciplinary working group (the Dutch Peritoneal Oncology Group [DPOG]), initiation of nationwide prospective studies, and the enhancement of referral networks. While a substantial body of literature emphasizes the reduction of inequalities in access to care, it primarily focuses on disparities driven by factors such as socioeconomic status (SES), race and ethnicity (e.g.,^10–12^). Despite numerous studies illustrating inter-hospital variation in access to care (e.g.,^6,7^), limited attention has been given to the effects of (national) efforts aimed at reducing such disparities. Hence, the current study assessed whether the variation in utilization of CRS-HIPEC, and, as a consequence, survival, of patients with isolated synchronous PMCRC reduced since national efforts were initiated.

## Methods

### Setting

Dutch hospitals can be divided into academic medical centers, teaching hospitals, and non-teaching hospitals.^13^ At the time of this study, there were eight expert centers in The Netherlands specializing in CRS-HIPEC. Expert centers are, by definition, academic or teaching hospitals, but academic and teaching hospitals are not always expert centers. Further information on the distribution and characteristics of Dutch hospitals treating CRC has been described elsewhere.^8^ Throughout the entire study period, CRS-HIPEC has been recognized as the standard of care in The Netherlands for patients presenting with limited isolated peritoneal metastases.^14^ CRS-HIPEC is exclusively performed by HIPEC centers and is conducted according to a nationwide protocol.^15^ Through the Health Insurance Act, all Dutch citizens are compulsorily insured for healthcare, making CRS-HIPEC reimbursable for all Dutch inhabitants if indicated.^13^

Several national initiatives have been undertaken since 2015. Notably, the DPOG was founded on 30 April 2015. Additionally, from 2017 onwards, a series of presentations and educational sessions were conducted to raise awareness regarding the observed disparities in the likelihood of undergoing CRS-HIPEC and, consequently, survival, as outlined in the publication by Rovers et al.^7^ These efforts were aimed at enhancing understanding about CRS-HIPEC as a treatment option and its specific indications. The period also witnessed the initiation of various prospective studies, including CAIRO6 (July 2017 to the present), COLOPEC (April 2015–February 2017), INTERACT (May 2018 to the present), and PIPAC-CRC (October 2017–September 2018).^16–19^ Lastly, the establishment of evolving referral networks has been a notable development over the years.

### Data Collection

This nationwide population-based cohort study used data from the Netherlands Cancer Registry (NCR)^20^ and was approved by the Scientific Committee of the Dutch CRS-HIPEC quality registry (K22.385).^21^ In the NCR, trained data managers extract data on patient, tumor, and treatment characteristics of all newly diagnosed malignancies in The Netherlands from the medical records. For vital status, an annual update is performed by linking the NCR to the Dutch municipal administrative database, which contains information on all current, deceased, and former residents of The Netherlands. Follow-up on vital status was available until 31 January 2023. Tumor location, histology, and staging were defined according to the International Classification of Diseases for Oncology, 3rd edition (ICD-O-3) and the Union for International Cancer Control (UICC) tumor-node-metastasis (TNM) classification according to the edition valid at the time of diagnosis, based on pathological stage, and supplemented with clinical stage if missing.^22,23^ Year of diagnosis was defined as the year of first histological confirmation, and hospital of diagnosis was defined as hospital of first contact for possible malignancy, whether outpatient or inpatient. Period of diagnosis was divided in 2009–2015 (i.e. before nationwide harmonization efforts) and 2016–2021 (i.e. during and after nationwide harmonization efforts). SES was based on individual fiscal data on the economic value of the home and household income, provided at an aggregated level per postal code. Treatment approach was categorized as CRS-HIPEC, systemic therapy, or other/no therapy. CRS-HIPEC could be performed with or without concomitant systemic therapy.^14^ Systemic therapy encompasses all chemotherapy and targeted therapy regimens with or without primary tumor resection or radiotherapy, but not in combination with CRS-HIPEC. OS was defined as the interval (in months) between diagnosis and death, or last follow-up date. For primary analysis, hospitals of diagnosis were classified as expert center or referring center; hospitals of diagnosis were further classified as academic/teaching hospital or non-teaching hospital.

### Patient Selection

This study included all adult patients (≥18 years) diagnosed with isolated synchronous peritoneal metastases of colorectal origin (C18-C20) in The Netherlands between 1 January 2009 and 31 December 2021. The following ICD-O-3 codes were considered peritoneal metastases: C16.0–C16.9, C17.0–C17.9, C18.0–C18.9, C19.9, C20.9, C21.8, C23.9, C26.9, C48.0–C48.8, C49.4–C49.5, C52.9, C54.3–C54.9, C55.9, C56.9, C57.0–C57.8, C66.9, C67.0–C67.9, C76.2. All other ICD-O codes were considered to be extraperitoneal metastases. Patients were excluded if the primary tumor was of appendiceal origin or concerned a neuroendocrine tumor.

### Analysis

Analyses were conducted using SAS^®^ 9.4 (SAS Institute, Inc., Cary, NC, USA). The significance level adopted was < 0.05. Univariate analyses were performed using Chi-square and Kruskal–Wallis tests where appropriate. Multilevel logistic regression models (2-level) were computed to assess the association between hospital of diagnosis and the odds of undergoing CRS-HIPEC while taking the leveled data structure into account (patients nested in hospitals).^24^ A Kenward-Roger correction was used to correct for the small effective sample sizes at hospital level.^25,26^ In a first model, an interaction term for hospital of diagnosis (referring center or expert center) with period of diagnosis was added to assess whether the interaction between hospital of diagnosis and the odds of undergoing CRS-HIPEC changed between periods. Given the significance of the interaction term (referring center with period, *p *< 0.001), the model was then run for both periods separately. Univariate survival distributions are presented as median OS with interquartile ranges (IQRs; in months) and the OS percentages. Multilevel Cox proportional hazard models were computed to assess the association between hospital of diagnosis (expert centers vs. referring centers) and survival while adjusting for potential confounders. For all multilevel analyses, random intercept models (at individual hospital level) with fixed effects were used. Variables included to correct for relevant case-mix factors were selected based on clinical relevance. Missing data, coded as ‘unknown’, were included in the analyses as separate dummies.

## Results

### Utilization of Cytoreductive Surgery-Hyperthermic Intraperitoneal Chemotherapy

**Fig. 1:**
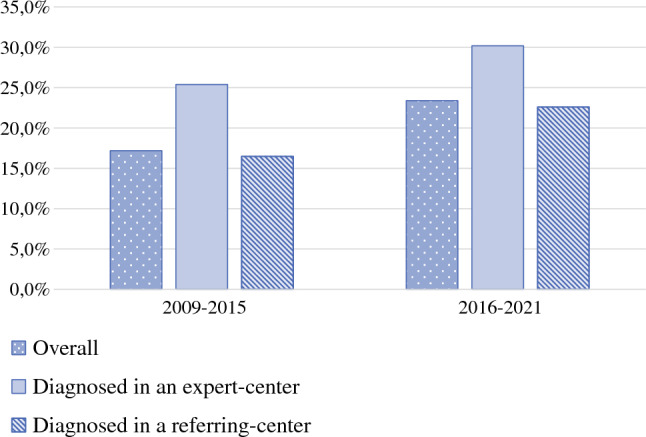
Proportion of patients who underwent cytoreductive surgery with hyperthermic intraperitoneal chemotherapy

A total of 3948 patients met the inclusion criteria and were included in this study, of whom 2151 (54.5%) were diagnosed in 2009–2015 (period 1), and 1797 (45.5%) were diagnosed in 2016–2021 (period 2). Of the 3948 included patients, 349 were diagnosed in an expert center (8.8%) and 3599 were diagnosed in a referring center (91.2%). In total, 20.0% of included patients underwent CRS-HIPEC, i.e. 27.8% of patients diagnosed in expert centers and 19.3% of patients diagnosed in referring centers (*p *< 0.001). The percentage of patients who underwent CRS-HIPEC significantly increased from 17.2% in period 1 (25.4% of patients diagnosed in expert centers vs. 16.5% of patients diagnosed in referring centers; *p *= 0.003) to 23.4% in period 2 (30.2% of patients diagnosed in expert centers versus 22.6% of patients diagnosed in referring centers; *p *= 0.025) [*p *< 0.001] (Fig. 1). Compared with patients diagnosed in referring centers, patients diagnosed in expert centers were younger and had a higher SES (Table 1).

**Table 1:** Baseline characteristics for patients diagnosed with isolated synchronous colorectal peritoneal metastases according to hospital of diagnosis being an expert center or referring center, stratified for period of diagnosis

	Overall (2009–2021)				Period 1 (2009–2015)				Period 2 (2016–2021)			
	All patients [*n* = 3948]	Diagnosis in referring center [*n* = 3599]	Diagnosis in expert center [*n* = 349]	*p*-Value	All patients [*n* = 2151]	Diagnosis in referring center [*n* = 1974]	Diagnosis in expert center [*n* = 177]	*p*-Value	All patients [*n* = 1797]	Diagnosis in referring center [*n* = 1625]	Diagnosis in expert center [*n* = 172]	*p*-Value
Hospital of diagnosis												
Non-teaching hospital	1586 (40.2)	1586 (44.1)	0 (0.0)	< 0.0001^a^	860 (40.0)	860 (43.6)	0 (0.0)	< 0.0001^a^	726 (40.4)	726 (44.7)	0 (0.0)	< 0.0001^a^
Academic/teaching hospital	2362 (59.8)	2013 (55.9)	349 (100.0)		1291 (60.0)	1114 (56.4)	177 (100.0)		1071 (59.6)	899 (55.3)	172 (100.0)	
Course of treatment												
CRS-HIPEC^c^	791 (20.0)	694 (19.3)	97 (27.8)	0.0004^a^	371 (17.2)	326 (16.5)	45 (25.4)	0.0070^a^	420 (23.4)	368 (22.6)	52 (30.2)	0.0696^a^
Systemic therapy^d^	1243 (31.5)	1153 (32.0)	90 (25.8)		761 (35.4)	710 (36.0)	51 (28.8)		482 (26.8)	443 (27.3)	39 (22.7)	
Other/no treatment	1914 (48.5)	1752 (48.7)	162 (46.4)		1019 (47.4)	938 (47.5)	81 (45.8)		895 (49.8)	814 (50.1)	81 (47.1)	
Year of diagnosis												
2009–2015	2151 (54.5)	1974 (54.8)	177 (50.7)	0.1389^a^								
2016–2021	1797 (45.5)	1625 (45.2)	172 (49.3)									
Sex												
Male	1890 (47.9)	1731 (48.1)	159 (45.6)	0.3648^a^	1048 (48.7)	962 (48.7)	86 (48.6)	0.9703^a^	842 (46.9)	769 (47.3)	73 (42.4)	0.2225^a^
Female	2058 (52.1)	1868 (51.9)	190 (54.4)		1103 (51.3)	1012 (51.3)	91 (51.4)		955 (53.1)	856 (52.7)	99 (57.6)	
Age at time of diagnosis, years												
Median (IQR)	71 (61–79)	71 (62–79)	68 (61–76)	0.0007^b^	70 (62–78)	70 (62–79)	68 (61–77)	0.0311^b^	71 (61–80)	72 (61–80)	69 (59–76)	0.0080^b^
Socioeconomic status												
Low	1017 (25.8)	936 (26.0)	81 (23.2)	0.0321^a^	697 (32.4)	640 (32.4)	57 (32.2)	0.4918^a^	320 (17.8)	296 (18.2)	24 (14.0)	0.0011^a^
Medium	1180 (29.9)	1087 (30.2)	93 (26.6)		838 (39.0)	775 (39.3)	63 (35.6)		342 (19.0)	312 (19.2)	30 (17.4)	
High	858 (21.7)	761 (21.1)	97 (27.8)		616 (28.6)	559 (28.3)	57 (32.2)		242 (13.5)	202 (12.4)	40 (23.3)	
Unknown	893 (22.6)	815 (22.6)	78 (22.3)						893 (49.7)	815 (50.2)	78 (45.3)	
T stage												
T0-3	1142 (28.9)	1049 (29.1)	93 (26.6)	0.1093^a^	597 (27.8)	557 (28.2)	40 (22.6)	0.2427^a^	545 (30.3)	492 (30.3)	53 (30.8)	0.0995^a^
T4	1941 (49.2)	1751 (48.7)	190 (54.4)		1082 (50.3)	984 (49.8)	98 (55.4)		859 (47.8)	767 (47.2)	92 (53.5)	
TX	865 (21.9)	799 (22.2)	66 (18.9)		472 (21.9)	433 (21.9)	39 (22.0)		393 (21.9)	366 (22.5)	27 (15.7)	
N stage												
N0	980 (24.8)	888 (24.7)	92 (26.4)	0.0531^a^	431 (20.0)	394 (20.0)	37 (20.9)	0.4882^a^	549 (30.6)	494 (30.4)	55 (32.0)	0.1679^a^
N1	1044 (26.4)	937 (26.0)	107 (30.7)		543 (25.2)	493 (25.0)	50 (28.2)		501 (27.9)	444 (27.3)	57 (33.1)	
N2	1248 (31.6)	1142 (31.7)	106 (30.4)		734 (34.1)	673 (34.1)	61 (34.5)		514 (28.6)	469 (28.9)	45 (26.2)	
NX	676 (17.1)	632 (17.6)	44 (12.6)		443 (20.6)	414 (21.0)	29 (16.4)		233 (13.0)	218 (13.4)	15 (8.7)	
Tumor location												
Colon	3542 (89.7)	3224 (89.6)	318 (91.1)	0.6578^a^	1913 (88.9)	1755 (88.9)	158 (89.3)	0.7692^a^	1629 (90.7)	1469 (90.4)	160 (93.0)	0.3508^a^
Rectosigmoid	87 (2.2)	80 (2.2)	7 (2.0)		39 (1.8)	37 (1.9)	2 (1.1)		48 (2.7)	43 (2.6)	5 (2.9)	
Rectum	319 (8.1)	295 (8.2)	24 (6.9)		199 (9.3)	182 (9.2)	17 (9.6)		120 (6.7)	113 (7.0)	7 (4.1)	
Tumor histology												
Adenocarcinoma	2441 (61.8)	2209 (61.4)	232 (66.5)	0.0960^a^	1306 (60.7)	1188 (60.2)	118 (66.7)	0.1539^a^	1135 (63.2)	1021 (62.8)	114 (66.3)	0.7400^a^
Mucinous carcinoma	892 (22.6)	829 (23.0)	63 (18.1)		554 (25.8)	519 (26.3)	35 (19.8)		338 (18.8)	310 (19.1)	28 (16.3)	
Signet ring cell carcinoma	400 (10.1)	369 (10.3)	31 (8.9)		223 (10.4)	207 (10.5)	16 (9.0)		177 (9.8)	162 (10.0)	15 (8.7)	
Unknown	215 (5.4)	192 (5.3)	23 (6.6)		68 (3.2)	60 (3.0)	8 (4.5)		147 (8.2)	132 (8.1)	15 (8.7)	
Tumor differentiation												
Good/moderately	1411 (35.7)	1285 (35.7)	126 (36.1)	0.4855^a^	700 (32.5)	645 (32.7)	55 (31.1)	0.7955^a^	711 (39.6)	640 (39.4)	71 (41.3)	0.4138^a^
Poorly/undifferentiated	897 (22.7)	810 (22.5)	87 (24.9)		528 (24.5)	481 (24.4)	47 (26.6)		369 (20.5)	329 (20.2)	40 (23.3)	
Unknown	1640 (41.5)	1504 (41.8)	136 (39.0)		923 (42.9)	848 (43.0)	75 (42.4)		717 (39.9)	656 (40.4)	61 (35.5)	

Data are expressed as *n* (%) unless otherwise specified

*CRS-HIPEC* cytoreductive surgery and hyperthermic intraperitoneal chemotherapy, *IQR* interquartile range

^a^ Chi-Square *p*-value

^b^ Kruskal–Wallis *p*-value

^c^ CRS-HIPEC could be performed with or without concomitant systemic therapy

^d^ Systemic therapy included all chemotherapy and targeted therapy regimens with or without primary tumor resection or radiotherapy, but without CRS-HIPEC

Multivariable analyses showed a significantly higher odds of undergoing CRS-HIPEC for patients diagnosed in expert centers compared with patients diagnosed in referring centers during period 1 (OR 1.64, 95% CI 1.02–2.67), whereas this difference was not observed in period 2 (OR 1.27, 95% CI 0.76–2.13) [Table 2]. In both time periods, there was no significant difference in the odds of undergoing CRS-HIPEC based on the teaching status of the hospital of diagnosis (OR 1.29, 95% CI 0.96–1.74 for period 1, and OR 1.13, 95% CI 0.82–1.56 for period 2) [Table 2].

**Table 2:** Multivariable multilevel regression analyses presenting the adjusted odds ratios for undergoing CRS-HIPEC in patients diagnosed with isolated synchronous colorectal peritoneal metastases in The Netherlands for period 1 (2009–2015) and period 2 (2016–2021)

	Period 1	Period 2
	(2009–2015)	(2016–2021)
	Adjusted OR (95% CI)	Adjusted OR (95% CI)
	[*n* = 2151]	[*n* = 1797]
Hospital of diagnosis		
Referring center	1.00	1.00
Expert center	**1.64 (1.02–2.67)**	1.27 (0.76–2.13)
Hospital of diagnosis		
Non-teaching hospital	1.00	1.00
Academic/teaching hospital	1.29 (0.96–1.74)	1.13 (0.82–1.56)
Sex		
Male	1.00	1.00
Female	1.00 (0.78–1.30)	1.07 (0.82–1.40)
Age, years	**0.93 (0.92–0.94)**	**0.94 (0.93–0.95)**
Socioeconomic status		
Low	1.00	1.00
Medium	1.21 (0.88–1.66)	1.09 (0.70–1.69)
High	1.27 (0.91–1.78)	1.47 (0.92–2.34)
Unknown		0.89 (0.61–1.31)
T stage		
T0-3	1.00	1.00
T4	**1.34 (1.01–1.78)**	**1.90 (1.41–2.55)**
TX	**0.08 (0.03–0.22)**	**0.19 (0.09–0.39)**
N stage		
N0	1.00	1.00
N1	0.95 (0.66–1.39)	**0.55 (0.39–0.80)**
N2	1.04 (0.77–1.41)	1.11 (0.81–1.51)
NX	**0.14 (0.06–0.30)**	**0.18 (0.07–0.45)**
Tumor location		
Colon	1.00	1.00
Rectosigmoid	0.96 (0.38–2.42)	1.43 (0.69–2.98)
Rectum	0.96 (0.62–1.49)	0.86 (0.50–1.46)
Tumor histology		
Adenocarcinoma	1.00	1.00
Mucinous carcinoma	**2.08 (1.54–2.82)**	**2.45 (1.75–3.42)**
Signet ring cell carcinoma	1.21 (0.75–1.96)	1.56 (0.93–2.60)
Unknown	0.43 (0.05–3.42)	0.42 (0.12–1.46)
Tumor differentiation		
Good/moderately	1.00	
Poorly/undifferentiated	**0.38 (0.27–0.54)**	**0.44 (0.31–0.62)**
Unknown	**0.55 (0.39–0.76)**	**0.25 (0.17–0.37)**

Statistically significant differences are indicated in bold

*OR* odds ratio, *CI* confidence interval, *CRS-HIPEC* cytoreductive surgery and hyperthermic intraperitoneal chemotherapy

### Survival

The median follow-up of all included patients was 10 months (IQR 3.1–23.3), and the 1-, 3-, and 5-year OS rates were 46.1%, 17.8%, and 10.2%, respectively (12.1% censored), with 1.824, 604, and 288 patients alive at these timepoints. The median OS of all included patients diagnosed between 2009 and 2021 was 10.4 months. This was 10.9 months for patients diagnosed in period 1, and 10.0 months for patients diagnosed in period 2 (*p *= 0.53).

In period 1, patients diagnosed in expert centers showed a significantly higher OS and lower hazard of death compared with patients diagnosed in referring centers (median OS 13.2 vs. 10.6 months; HR 0.80, 95% CI 0.66–0.96). This significant difference was no longer present in period 2 (median OS 13.0 vs. 9.8 months; HR 1.00, 95% CI 0.76–1.32). Patients undergoing CRS-HIPEC showed a higher OS, with a significantly lower hazard of death in both periods (Table 3). Other variables that showed a negative impact on survival were older age, higher or unknown tumor T and N stage, and poorly or undifferentiated tumors. The tumor histology being a mucinous carcinoma significantly lowered the hazard of death. A high SES was associated with a lower hazard of death in the period 2016–2021 only (Table 3).

**Table 3:** Univariate and multilevel multivariable Cox proportional hazard analysis for determining predictors of overall survival in patients diagnosed with isolated synchronous colorectal peritoneal metastases in The Netherlands between 2009–2015 and 2016–2021

Variable	OS in months	3-year OS	Multivariable analysis^a^	OS, months	3-year OS	Multivariable analysis^a^
	Period 1	Period 1	Period 1	Period 2	Period 2	Period 2
	(2009–2015)	(2009–2015)	(2009–2015	(2016–2021)	(2016–2021)	(2016–2021)
			[*n* = 2140]			[*n* = 1784]
	Median (IQR)	%	HR (95%CI)	Median (IQR)	%	HR (95%CI)
Hospital of diagnosis						
Expert center	13.2 (5.0–31.2)	23.7	**0.80 (0.66–0.96)**	13.0 (3.1–31.7)	22.1	1.00 (0.76–1.32)
Referring center	10.6 (3.5–25.8)	16.7	1.00	9.8 (2.6–25.2)	18.0	1.00
Hospital of diagnosis						
Non-teaching	10.5 (3.4–24.8)	15.4	1.00	9.4 (2.5–24.9)	17.0	1.00
Academic/teaching	11.2 (3.6–28.0)	18.5	0.97 (0.88–1.08)	10.2 (2.8–26.5)	19.4	0.92 (0.78–1.09)
Course of treatment						
CRS-HIPEC^b^	36.7 (20.5–69.6)	49.9	1.00	36.2 (19.5–69.1)	50.3	1.00
Systemic therapy^c^	13.0 (7.0–26.2)	14.7	**1.72 (1.49–1.99)**	12.8 (7.5–22.2)	12.0	**1.83 (1.54–2.18)**
Other/no treatment	4.4 (1.3–12.4)	7.2	**3.73 (3.21–4.35)**	3.0 (1.0–9.7)	6.6	**3.86 (3.26–4.57)**
Sex						
Male	10.5 (3.7–25.0)	16.3	1.00	9.7 (2.6–25.3)	16.1	1.00
Female	11.3 (3.3–28.3)	18.1	0.92 (0.84–1.00)	10.2 (2.8–26.4)	20.5	0.93 (0.83–1.03)
Age, years						
Median = 71			1.01 (1.00–1.01)			**1.01 (1.01–1.02)**
Socioeconomic status						
Low	9.6 (2.9–24.2)	15.2	1.00	8.4 (2.3–20.6)	15.6	1.00
Middle	11.3 (3.6–26.9)	17.4	0.99 (0.89–1.10)	10.4 (3.0–25.8)	17.8	0.93 (0.79–1.10)
High	11.9 (4.2–28.4)	19.2	1.00 (0.89–1.12)	15.2 (4.5–38.2)	26.0	**0.81 (0.67–0.98)**
Unknown				9.4 (2.5–25.3)	17.0	1.00 (0.86–1.15)
T stage						
T0-3	18.2 (7.1–41.7)	27.8	1.00	10.3 (3.1–26.1)	19.2	1.00
T4	12.5 (4.8–27.6)	18.5	**1.54 (1.38–1.72)**	14.0 (4.6–35.9)	24.8	1.07 (0.95–1.22)
Tx	3.8 (1.3–9.3)	0.01	**2.83 (2.42–3.32)**	3.1 (1.0–10.8)	0.03	**1.47 (1.26–1.72)**
N stage						
N0	11.6 (3.6–35.3)	24.4	1.00	8.6 (2.1–22.2)	16.5	1.00
N1	15.0 (5.6–33.8)	23.2	1.18 (1.03–1.35)	14.6 (4.9–36.2)	25.0	0.95 (0.82–1.10)
N2	13.5 (6.3–28.0)	18.1	**1.37 (1.21–1.57)**	12.7 (4.9–31.4)	20.8	1.09 (0.94–1.26)
NX	3.5 (1.3–10.8)	1.1	**1.74 (1.50–2.03)**	2.3 (0.5–6.9)	2.8	**1.79 (1.51–2.13)**
Primary tumor location						
Colon	10.8 (3.3–26.2)	17.5	1.00	9.9 (2.6–25.3)	18.0	1.00
Rectosigmoid	13.0 (4.7–30.9)	10.3	1.18 (0.85–1.64)	13.0 (3.7–35.8)	22.9	1.19 (0.87–1.64)
Rectum	11.6 (5.8–22.7)	15.6	**1.21 (1.03–1.42)**	10.9 (4.0–32.4)	21.0	0.94 (0.76–1.16)
Tumor histology						
Adenocarcinoma	11.5 (3.6–29.0)	18.8	1.00	10.3 (3.0–27.5)	19.7	1.00
Mucinous carcinoma	12.9 (5.0–27.6)	19.5	0.90 (0.81–1.01)	16.3 (6.0–39.5)	25.9	**0.83 (0.72–0.96)**
Signet ring cell carcinoma	8.6 (3.1–16.6)	5.8	**1.28 (1.10–1.49)**	9.4 (2.7–18.3)	8.2	0.91 (0.75–1.10)
Other	1.3 (0.5–5.1)	2.9	**1.47 (1.14–1.91)**	1.0 (0.4–4.9)	2.7	**1.57 (1.28–1.93)**
Tumor differentiation						
Well/moderately	21.8 (9.4–43.6)	30.6	1.00	19.3 (7.1–45.5)	31.0	
Poorly/undifferentiated	8.7 (3.4–19.8)	12.5	**1.63 (1.44–1.84)**	8.8 (3.0–21.6)	14.8	**1.49 (1.29–1.74)**
Unknown	6.9 (2.1–17.1)	9.8	**1.70 (1.51–1.92)**	4.8 (1.0–13.9)	7.7	**1.81 (1.57–2.08)**

Statistically significant differences are indicated in bold

*OS* overall survival, *IQR* interquartile range, *CI* confidence interval, *CRS-HIPEC* cytoreductive surgery with hyperthermic intraoperative intraperitoneal chemotherapy

^a^ Multilevel Cox proportional hazard model with random intercept and fixed effects

^b^ CRS-HIPEC could be performed with or without concomitant systemic therapy

^c^ Systemic therapy included all chemotherapy and targeted therapy regimens with or without primary tumor resection or radiotherapy, but without CRS-HIPEC

## Discussion

This nationwide population-based study assessed whether the previous existing variation in the utilization of CRS-HIPEC for synchronous PMCRC decreased after several national initiatives were implemented. This study found that the previously observed variation based on hospital of diagnosis no longer existed in the period 2016–2021. Furthermore, with regard to survival, the significant difference in hazard of death as observed in the initial period (2009–2015) decreased, leading to similar survival outcomes for patients diagnosed in referring centers as compared with patients diagnosed in expert centers in the latter period (2016–2021).

The provision of a certain specialized treatment is hospital-dependent; a hospital either offers a specific type of care or it does not, making specialized treatment an institutional resource. Therefore, patient accessibility to this treatment could be influenced by the hospital in which the diagnosis was made.^27^ Besides, other factors on the hospital level could influence treatment variation, such as hospital type,^28^ which is a proxy for other factors such as the presence of expertise multidisciplinary team meetings (MDTs), physician’s experiences,^29^ and treatment preferences.^30–32^ Several options for reducing this institutional variation have been suggested, such as the dissemination of reliable information among physicians,^33^ establishing well-defined clinical recommendations,^33^ and setting up regional referral networks with MDTs.^34^ In the time following the previous publication,^7^ several of these options have been observed in The Netherlands. For example, a national multidisciplinary working group on PMCRC and CRS-HIPEC was established to offer guideline recommendations and to enhance referral networks for patients with PMCRC, to ensure that patients receive appropriate treatment and seamless referrals to expert centers when needed. Furthermore, multiple presentations were held at both national and international conferences to discuss previous findings, thereby enhancing the awareness of CRS-HIPEC as a treatment option for PMCRC. The awareness was also increased because several national prospective trials were initiated (e.g., CAIRO6, COLOPEC, INTERACT, and PIPAC-CRC^16–19^), which have arguably further fueled the conversation and discussion about potential treatment options for patients with PMCRC, potentially resulting in more patients being discussed at regional MDT meetings. These initiatives are primarily focused on improving referral patterns and increasing the knowledge and expertise of medical specialists concerning CRS-HIPEC and its implications. One possible cause for the differences found in the initial period is that not every patient who may be eligible for CRS-HIPEC is referred to a HIPEC center. In 2015, a Dutch nationwide study showed that half of medical oncologists involved in colorectal cancer, and a quarter of the surgeons involved in colorectal cancer, did not consider CRS-HIPEC as standard care for patients with limited isolated PMCRC.^30^ Moreover, an American survey study among physicians showed that survival after CRS-HIPEC was often underestimated, while 30-day mortality was overestimated by more than half of respondents, and that almost half of physicians would not refer a patient due to lack of access to HIPEC centers.^35^ Therefore, the lack of awareness and knowledge about the implications and potential positive effects of CRS-HIPEC, along with the absence of proper referral pathways, could possibly explain a significant portion of the non-referred patients in the period 2009–2015. The dissolving of this previously observed variation in utilization of CRS-HIPEC may thus be explained by efforts to facilitate the referral of patients and to improve the familiarity of physicians with this low-volume, high-complexity disease entity.

In addition to the efforts specifically aimed at caring for PMCRC, in 2014 the population screening program for colorectal carcinoma was introduced in The Netherlands.^36,37^ This program has led to the earlier diagnosis of patients.^36^ As a result, patients might be identified at a less advanced stage, with less extensive peritoneal metastases, i.e., lower Peritoneal Cancer Index (PCI) scores. This could potentially make more patients eligible for CRS-HIPEC, which may explain a part of the increase in the percentage of patients undergoing CRS-HIPEC. However, the population screening program was implemented nationwide and its potential impact applies to both the referring centers and expert centers.

In accordance with the study by Rovers et al.^7^ patients diagnosed in expert centers between 2009 and 2015 had a significantly lower hazard of death compared with patients diagnosed in referring centers. This difference disappeared in the period 2016–2021 and is accompanied with similar survival outcomes for patients diagnosed in referring centers as compared with patients diagnosed in expert centers. The treatment effect of CRS-HIPEC thus appears to have a favorable impact on survival when compared with systemic therapy alone or other/no treatment in both time periods. However, the treatment course does not seem to account for the entire observed survival variation between referring centers and expert centers between 2009 and 2015. Despite adjusting for treatment, the survival difference between referring centers and expert centers that was evident from 2009 to 2015 disappeared by 2016–2021. This suggests that the reduction in variability in the odds of undergoing CRS-HIPEC may have influenced the disparity in survival outcomes. The reduced variation in the utilization of CRS-HIPEC therefore also appears to be reflected in a reduced variation in survival between hospitals of diagnosis. Although the multivariable analysis shows a decrease in survival differences between hospitals of diagnosis in the period 2016–2021 compared with the period 2009–2015, the absolute OS has not increased. This can be explained by the fact that the majority of patients with synchronous PMCRC receive systemic therapy or no treatment at all. These patients, who generally have a poor survival, largely influence the OS outcome.

Several previous studies have focused on unequal access to specialized, centralized care based on hospital type, such as the study by Rovers et al. on PMCRC,^7^ as well as studies on pancreatic, esophageal, gastric, and liver cancer surgery.^34,38,39^ The findings from the period 2009–2015 in this study are mainly in line with the previous study by Rovers et al. on variation in utilization of CRS-HIPEC for PMCRC.^7^ However, Rovers et al. also found an independent significant association between the teaching status of the hospital of diagnosis and the odds of undergoing CRS-HIPEC. The absence of this association in this current study is probably explained by the statistical approach adopted. In contrast to other studies on the variation in healthcare utilization, the current study employs multilevel analyses to address the nested data structure, which is a more conservative approach. Despite the use of a correction factor for small sample sizes at the hospital level, this approach results in somewhat wider CIs.

Limitations of this study can be found in its observational nature. For example, no data were available on comorbidities, performance status and extent of peritoneal disease. Patient's overall health and the extent of the disease are, among other factors, often utilized to determine whether a specific treatment is offered. Consequently, these factors may have played a role in the likelihood of undergoing CRS-HIPEC and subsequent OS, potentially leading to a confounding effect if not evenly distributed between the two groups (patients diagnosed in HIPEC centers vs. referring centers). However, given the population-based nature of this study with national coverage, it is expected that these potential confounders are evenly distributed across the groups. Moreover, the potential impact of this confounding effect applies to both the period before the initiation of national efforts and the period after, therefore not explaining the decrease in interhospital variation. Furthermore, only data on performed treatment were available, but not on intended treatment. However, the population-based nature of the study let us expect that this would also be equally distributed between patients from different hospitals. Furthermore, patients with metachronous PMCRC were not included due to data availability, since, for the timeframe included in this study, the NCR only contains systematically recorded data on synchronous metastases. However, based on clinical reasoning, it is assumed that these results also apply to patients with metachronous PMCRC. Because of its observational nature, this study reveals a significant association between the national efforts implemented and the decrease in variation in odds of undergoing CRS-HIPEC and subsequent survival, rather than establishing causation. Nonetheless, these results could set an example for both other countries and other disciplines, as obtaining an overview of the treatment variation within a country, as well as combining education and cooperation, seems to contribute to the elimination of treatment variation based on hospital of diagnosis. This enables improved and more equal treatment results for patients, indicating the importance of these population-based data studies.

## Conclusion

Variation in the likelihood of receiving specific treatment should not depend on the hospital of diagnosis, as this possibly causes patients to miss out on life-extending or even curative treatment options. This study observed significant differences in the likelihood of undergoing CRS-HIPEC, and subsequently survival, between 2009 and 2015, which, in a period of national efforts to harmonize the treatment for patients with PMCRC, were eliminated. The previously observed inequality in the odds of undergoing CRS-HIPEC, as well as the difference in survival, based on the hospital of diagnosis, was no longer present between 2016 and 2021 in The Netherlands, leading to similar survival outcomes for patients diagnosed in referring centers as compared with patients diagnosed in expert centers. This study emphasizes the value of observational population-based data for understanding and addressing treatment and survival differences within a country, and suggests that education, cooperation, and the establishment of referring networks could contribute to the elimination of undesirable practice variation. These findings might serve as an example for both other nations and other disciplines, hoping to reduce interhospital practice variation for a broader population, thereby increasing patients’ chances of treatment and subsequent survival.

## Data Availability

The data that support the findings of this study are available from the NCR, maintained by the Netherlands Comprehensive Cancer Organization. Restrictions apply to the availability of these data, which were used under license for this study. Data are available with the permission of the Netherlands Comprehensive Cancer Organization.
